# 1777. Yellow Fever Dynamics in Ghana During the Past Century

**DOI:** 10.1093/ofid/ofad500.1606

**Published:** 2023-11-27

**Authors:** Seth D Judson, David W Dowdy, Franklin Asiedu-Bekoe, Alberta Biritwum-Nyarko, Ernest Kenu, Lee Schroeder

**Affiliations:** Johns Hopkins University School of Medicine, Baltimore, Maryland; Johns Hopkins Bloomberg School of Public Health, Baltimore, Maryland; Ghana Health Service, Accra, Greater Accra, Ghana; Ghana Health Service, Accra, Greater Accra, Ghana; University of Ghana, Accra, Greater Accra, Ghana; University of Michigan, Ann Arbor, Michigan

## Abstract

**Background:**

Although West Africa has the largest burden of Yellow Fever (YF) cases globally, less is known about YF dynamics in this region. YF virus is thought to circulate via three transmission cycles in Africa: the sylvatic, savannah/intermediate, and urban cycles. Understanding the relative contribution of each cycle and the populations at risk could inform allocation of YF diagnostics and vaccines. Therefore, we analyzed the history of YF in Ghana over the past century to inform preparedness for future outbreaks.

**Methods:**

We reviewed the primary literature for all YF outbreaks in Ghana as well as WHO YF documents, including the Disease Outbreak News and Weekly Epidemiology Record. We investigated the epidemiology of each outbreak to determine the most likely regions and populations involved. We also reviewed the epidemiology, vectors, and seasonality of outbreaks to determine the most likely cycles.

**Results:**

From 1910-2022 there were 2378 cases and 872 deaths reported due to YF in Ghana (Figure). The first confirmed outbreak of YF in Ghana occurred in 1910, and for 50 years the majority of outbreaks occurred in coastal regions via the urban cycle, with outbreaks beginning early in the year during the dry season. Following this period, there were large outbreaks of urban YF in multiple regions (including the Upper East, Upper West, Eastern, and Volta regions). In 1992 childhood YF immunization became routine in Ghana and smaller outbreaks occurred in northern regions where immunization was less complete. Twelve reactive vaccination campaigns occurred from 1951-2021. Since 2005, outbreaks of YF in Ghana have been due to the sylvatic/savannah cycles. A mass preemptive vaccination campaign was conducted in 2020, followed by a sylvatic/savannah outbreak in 2021 among largely unvaccinated nomadic groups.

**Figure:**
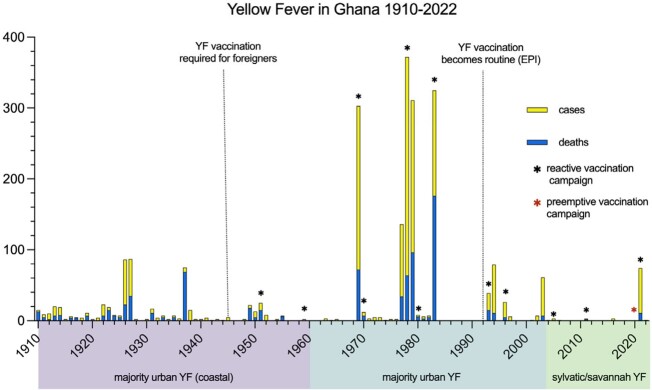

**Conclusion:**

The dynamics of YF in Ghana have changed over the past century. Initial outbreaks of YF in Ghana were characterized by the urban cycle. Following multiple vaccination campaigns, recent outbreaks reflect the sylvatic and savannah cycles. Regions currently at highest risk for future YF outbreaks are those with unvaccinated populations and suitable habitats for the vectors and non-human primates that propagate the sylvatic and savannah cycles

**Disclosures:**

**All Authors**: No reported disclosures

